# Supplementary figures and images for: In silico design of a multi-epitope vaccine against Cryptosporidium parvum using structural and immunoinformatics approaches
Source: PLoS One. 2025 Nov 18;20(11):e0334754. doi: 10.1371/journal.pone.0334754 (PMC12626319; doi:10.1371/journal.pone.0334754)

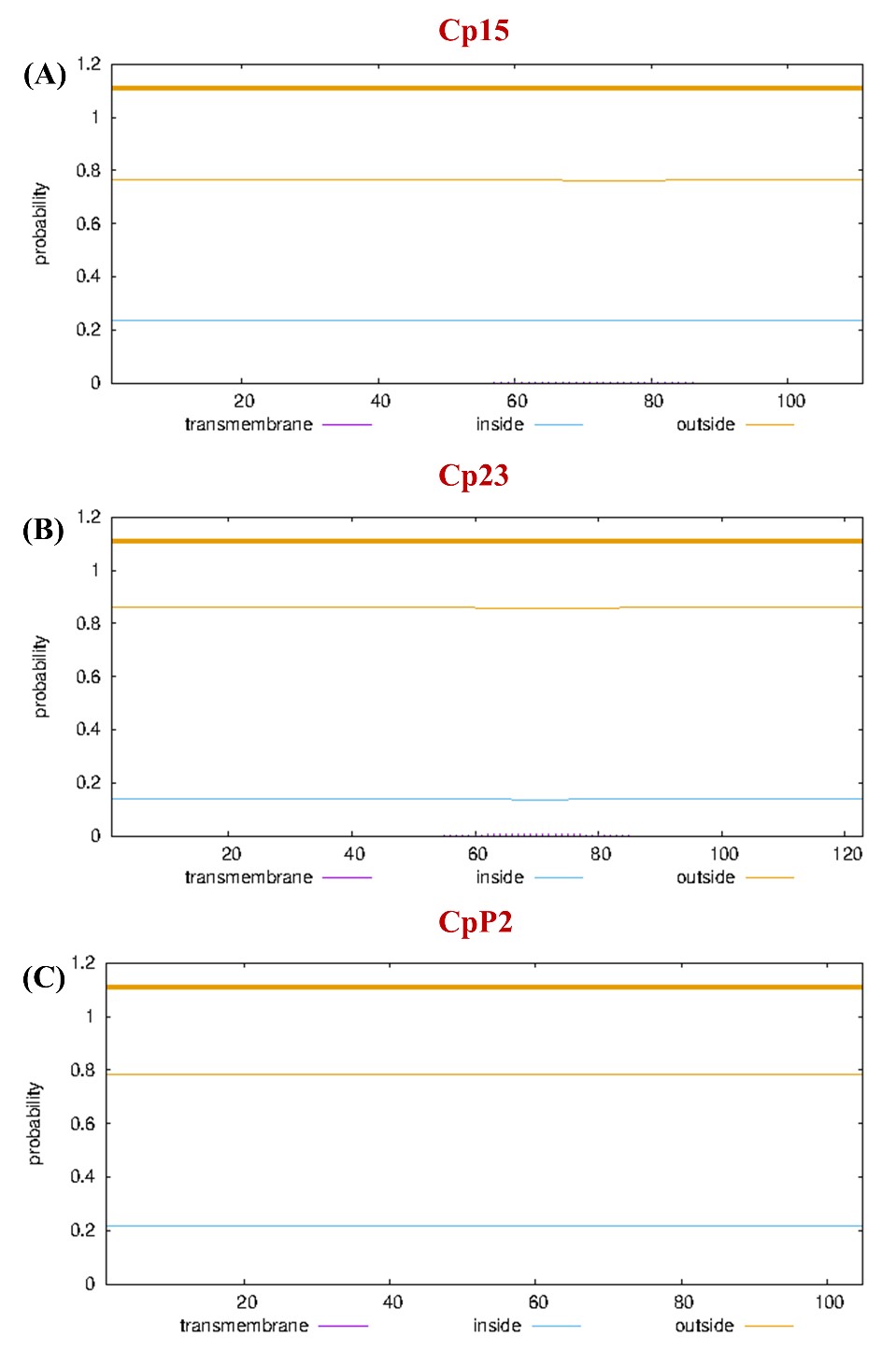

Supplement: S1 Fig — (A) Cp15, (B) Cp23, and (C) CpP2. The x-axis represents the amino acid position in the sequence, and the y-axis indicates the probability of a transmembrane helix at that position. (JPG) [file pone.0334754.s001.jpg]

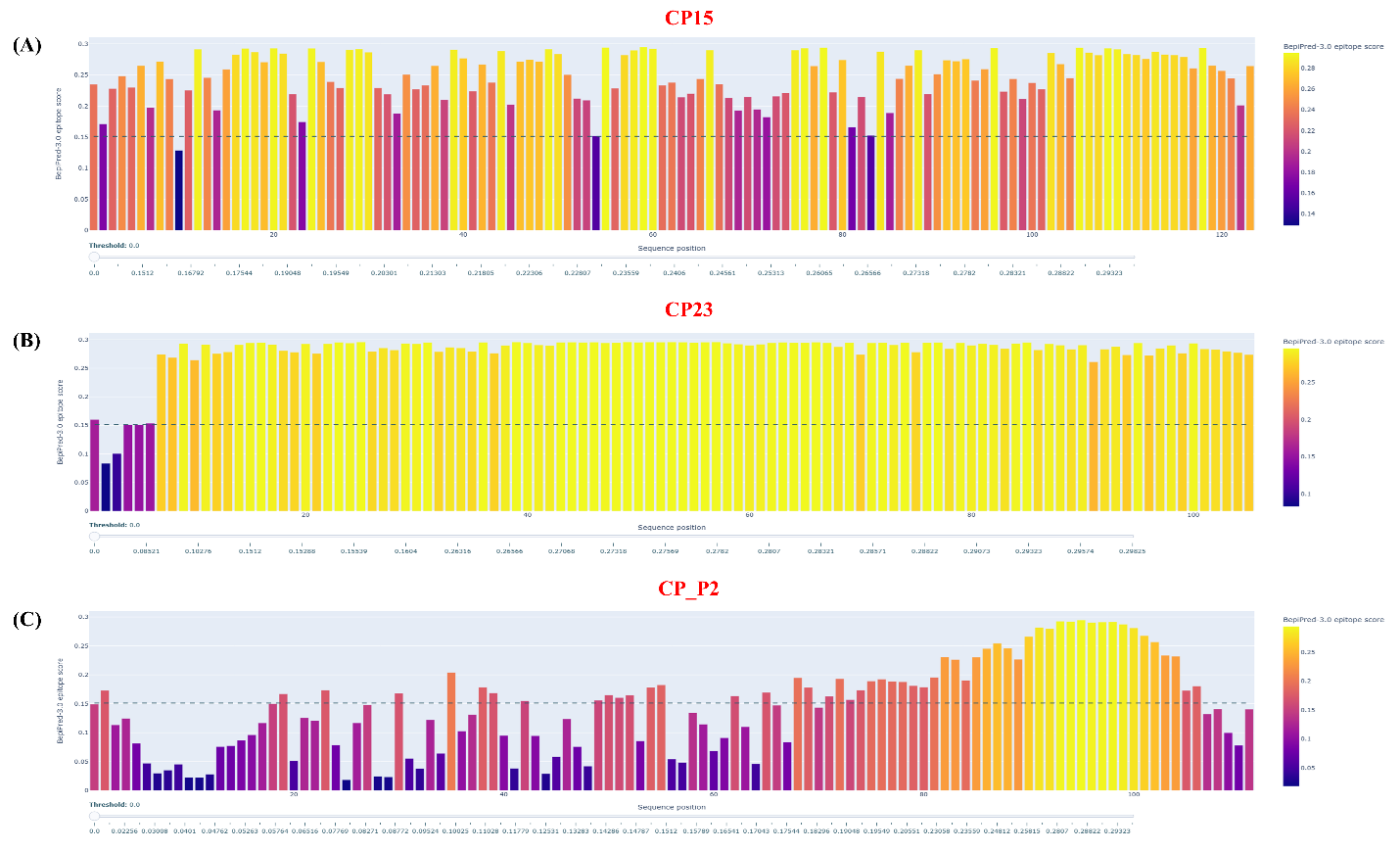

Supplement: S2 Fig — (A) Cp15, (B) Cp23, and (C) CpP2. The Y-axis indicates the epitope prediction score, and the X-axis represents the amino acid position within each protein. Regions above the threshold line represent predicted linear B-cell epitopes. (TIF) [file pone.0334754.s002.tif]

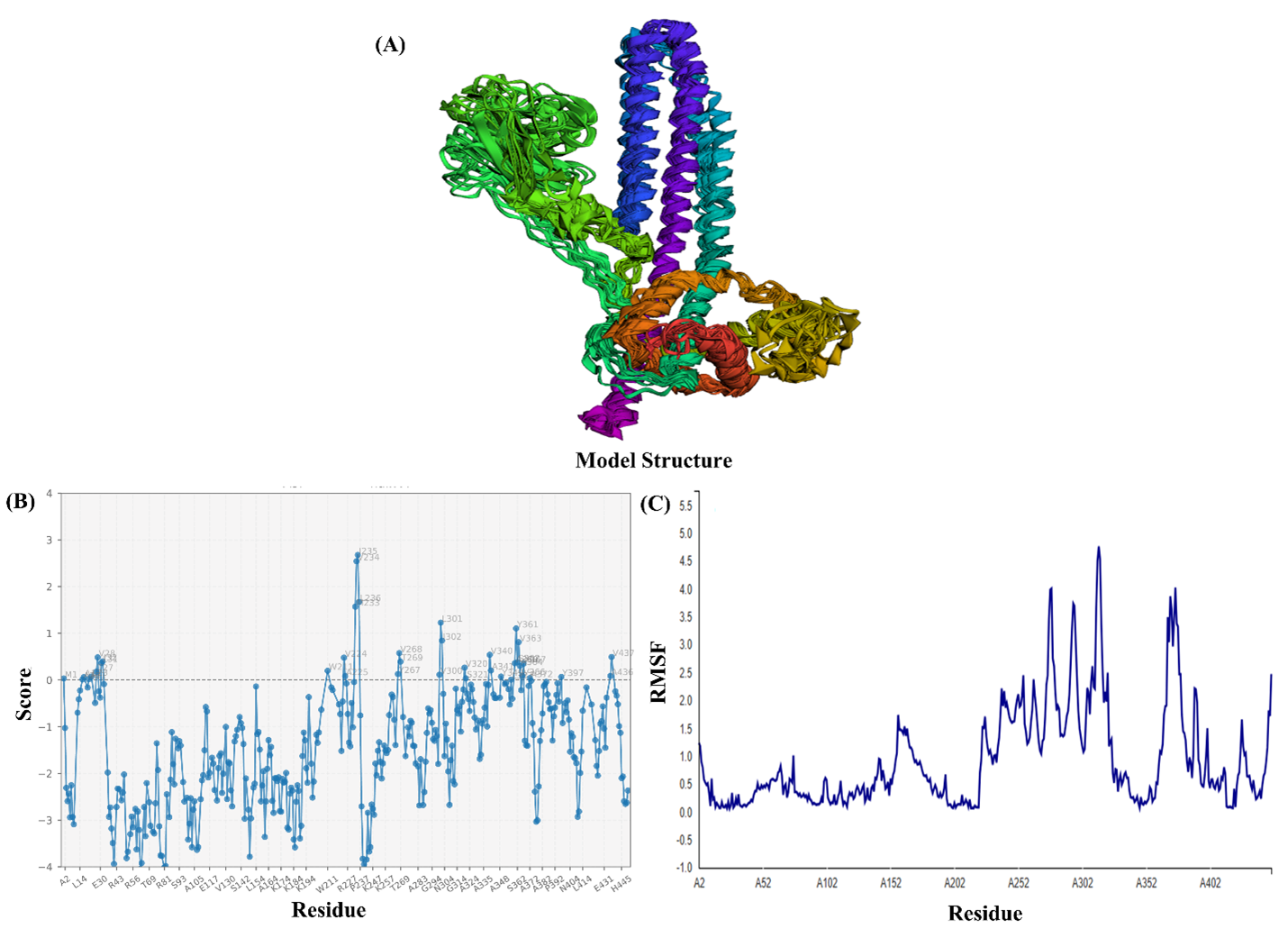

Supplement: S3 Fig — (A) Predicted structural models generated through CABS-flex analysis. (B) AGGRESCAN output showing residues with scores greater than zero, indicating their propensity for aggregation. (C) Flexibility profile from CABS-flex, where residue fluctuations are represented as RMSF values. (TIF) [file pone.0334754.s003.tif]

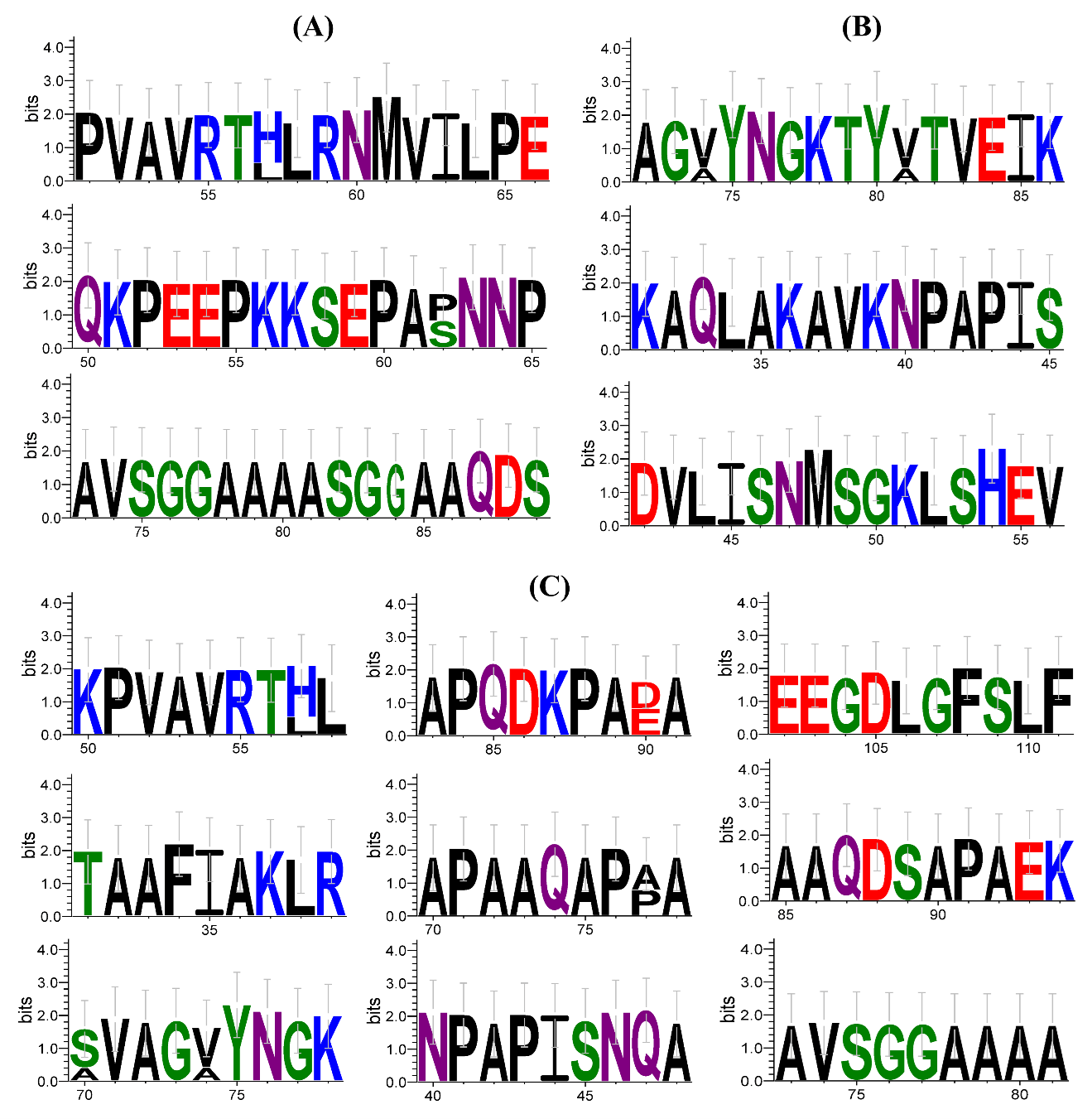

Supplement: S4 Fig — Sequence logo visualization of predicted epitopes from (A) Cp15, (B) Cp23, and (C) CpP2 proteins. The x-axis denotes the position of amino acids within the epitope sequences, while the y-axis shows the relative frequency of each residue at those positions. The overall height of each letter stack indicates the degree of conservation, with taller stacks representing higher conservation across aligned sequences. (TIF) [file pone.0334754.s004.tif]

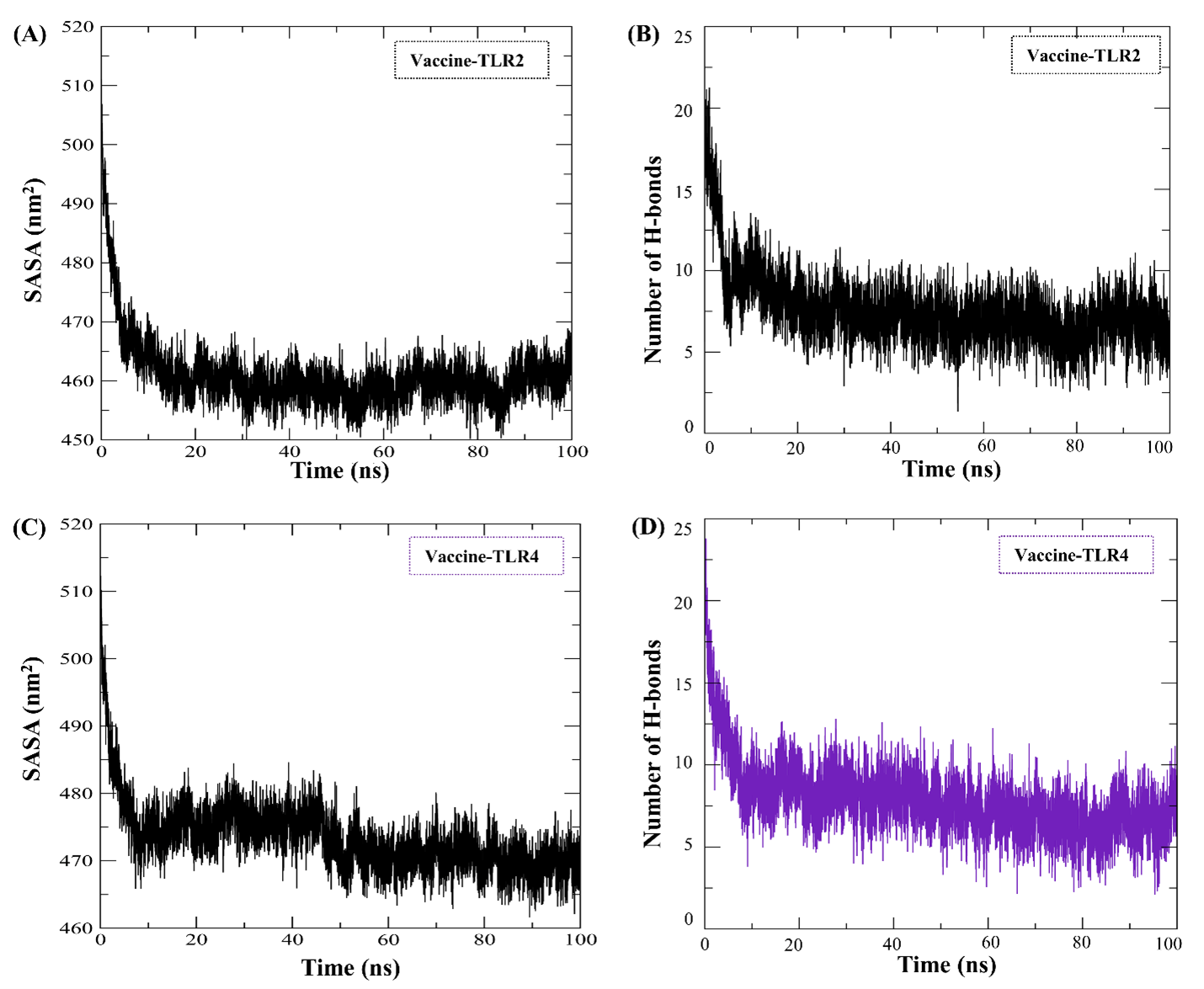

Supplement: S5 Fig — (A) SASA plot of the vaccine–TLR2 complex (black); (B) Hb plot of the vaccine–TLR2 complex (black); (C) SASA plot of the vaccine–TLR4 complex (black); (D) Hb plot of the vaccine–TLR4 complex (purple). (TIF) [file pone.0334754.s005.tif]
