# Supplementary material for: In silico design of a multi-epitope vaccine against Cryptosporidium parvum using structural and immunoinformatics approaches
Source: PLoS One. 2025 Nov 18;20(11):e0334754. doi: 10.1371/journal.pone.0334754 (PMC12626319; doi:10.1371/journal.pone.0334754)
Supplement: S1 Table — (DOCX) [file pone.0334754.s006.docx]

**S1 Table**. Overview of databases and web-based tools employed in the design of a multi-epitope subunit vaccine against *C. parvum.*

| **Database, software, or web service** | **Description** | **Uniform Resource Locator (URL)** |
| --- | --- | --- |
| UniProt | A resource of protein sequences and functional information | <https://www.uniprot.org/> |
| VaxiJen v2.0 | Prediction of antigenicity | <https://www.ddg-pharmfac.net/vaxijen/VaxiJen/VaxiJen.html> |
| AllerCatPro 2.0 | Prediction of allergenicity | https://allercatpro.bii.a-star.edu.sg/ |
| BLASTp | Compares a protein query to a protein database | <https://blast.ncbi.nlm.nih.gov/>  [Blast.cgi?PROGRAM=blastp&PAGE_TYPE=](https://blast.ncbi.nlm.nih.gov/)  [BlastSearch&LINK_LOC=blasthome](https://blast.ncbi.nlm.nih.gov/) |
| TMHMM- 2.0 | Prediction of transmembrane helices in proteins | https://services.healthtech.dtu.dk/services/TMHMM-2.0/ |
| ABCpred | Prediction of B-cell epitope | <http://crdd.osdd.net/raghava/abcpred/> |
| BepiPred-3.0 | Prediction of potential B-cell epitopes from protein sequence | https://services.healthtech.dtu.dk/services/BepiPred-3.0/ |
| IEDB MHC II | Prediction of helper T-lymphocyte epitope | <https://tools.iedb.org/mhcii/> |
| IFNepitope | Predicting and designing  interferon-gamma inducing epitopes | <http://crdd.osdd.net/raghava/ifnepitope/predict.php> |
| IL4Pred | Prediction of IL4-inducing peptides | <https://webs.iiitd.edu.in/raghava/il4pred/predict.php> |
| IEDB MHC-I server | T Cell Prediction - Class I | https://nextgen-tools.iedb.org/pipeline?tool=tc1 |
| ToxinPred | Prediction of toxicity | <https://webs.iiitd.edu.in/raghava/toxinpred/> |
| ProtParam | Physicochemical properties | <https://web.expasy.org/protparam/> |
| AllerTop v.2.0 | Prediction of allergenicity | https://www.ddg-pharmfac.net/allertop_test/ |
| ANTIGENpro | Prediction of antigenicity | <https://scratch.proteomics.ics.uci.edu/> |
| Protein-Sol | Solubility prediction | https://protein-sol.manchester.ac.uk/ |
| SOLpro | Solubility prediction | https://scratch.proteomics.ics.uci.edu/ |
| PSIPRED v4.0 | Secondary structure prediction | <http://bioinf.cs.ucl.ac.uk/psipred/> |
| GOR IV | Secondary structure prediction | <https://npsa-prabi.ibcp.fr/cgi-bin/npsa_automat.pl?page=/NPSA/npsa_gor4.html> |
| trRosetta | Protein structure and function Prediction | <http://raptorx6.uchicago.edu/> |
| GalaxyRefine2 | Protein structure refinement | https://galaxy.seoklab.org/cgi-bin/submit.cgi?type=REFINE2 |
| PROCHECK | Protein structure  validation | <https://saves.mbi.ucla.edu/> |
| ProSA-web | Protein structure  validation | <https://prosa.services.came.sbg.ac.at/prosa.php> |
| Chimera 1.17.1 | Interactive visualization and analysis of molecular structure | <https://www.rbvi.ucsf.edu/chimera/> |
| CABS-flex 2.0 | Structural flexibility analysis | https://biocomp.chem.uw.edu.pl/CABSflex2 |
| AGGRESCAN 3D server v2.0 | To identify aggregation-prone regions | https://biocomp.chem.uw.edu.pl/A3D2/ |
| IEDB ElliPro | Antibody Epitope Prediction | <http://tools.iedb.org/ellipro/> |
| IEDB population coverage | Population coverage prediction | <http://tools.iedb.org/population/> |
| ClusPro 2.0 | Docking study | <https://cluspro.bu.edu/login.php> |
| PDBsum | Structural interaction analysis | http://www.ebi.ac.uk/pdbsum |
| GROMACS | A molecular dynamic package mainly designed for simulations of proteins, lipids, and nucleic acids | <https://www.gromacs.org/> |
| HawkDock | A tool for MM-GBSA calculation | <https://cadd.zju.edu.cn/hawkdock/> |
| C-ImmSim | Immune simulation | <https://kraken.iac.rm.cnr.it/C-IMMSIM/index.php> |
| Java Codon Adaptation Tool | Codon optimization | https://jcat.de/ |
| SnapGene | *In-silico* simulation | <https://www.snapgene.com/> |
| RNAfold web server | Predict secondary structures of single-stranded RNA or DNA sequences. | http://rna.tbi.univie.ac.at/cgi-bin/RNAWebSuite/RNAfold.cgi |
