# Supplementary material for: In silico design of a multi-epitope vaccine against Cryptosporidium parvum using structural and immunoinformatics approaches
Source: PLoS One. 2025 Nov 18;20(11):e0334754. doi: 10.1371/journal.pone.0334754 (PMC12626319; doi:10.1371/journal.pone.0334754)
Supplement: S3 Table — (DOCX) [file pone.0334754.s008.docx]

**S3 Table.** Prediction of linear (continuous) antibody epitopes using the ElliPro server.

| **Si. No.** | **Start** | **End** | **Peptide** | **Residues** | **Score** |
| --- | --- | --- | --- | --- | --- |
| 1 | 266 | 306 | TYVTVEIKGPGPGKAQLAKAVKNPAPISGPGPGDVLISNMS | 41 | 0.766 |
| 2 | 58 | 89 | TKLQEDLPEQLTELREKFTAEELRKAAEGYLE | 32 | 0.746 |
| 3 | 138 | 181 | GTVASQTRAVGERAAKLVGIELPKKAAPAKKAAPAKKAAPAKKA | 44 | 0.744 |
| 4 | 358 | 391 | RAAYSVAGVYNGKAAYAPQDKPAEAAAYAPAAQA | 34 | 0.744 |
| 5 | 220 | 263 | AAKPVAVRTHLRNMVILPEKKQKPEEPKKSEPASNNPKKAGVYN | 44 | 0.638 |
| 6 | 440 | 450 | GAAAAHHHHHH | 11 | 0.613 |
| 7 | 424 | 437 | AQDSAPAEKAAYAV | 14 | 0.559 |
| 8 | 335 | 340 | AAYKPV | 6 | 0.524 |
