# Supplementary material for: In silico design of a multi-epitope vaccine against Cryptosporidium parvum using structural and immunoinformatics approaches
Source: PLoS One. 2025 Nov 18;20(11):e0334754. doi: 10.1371/journal.pone.0334754 (PMC12626319; doi:10.1371/journal.pone.0334754)
Supplement: S4 Table — (DOCX) [file pone.0334754.s009.docx]

**S4 Table.** ElliPro-based prediction of conformational (discontinuous) antibody epitopes.

| **Si. No.** | **Discontinuous epitope no.** | **No. of residues** | **Score** |
| --- | --- | --- | --- |
| **1** | Q241, K242, P243, E244, E245, P246, K247, K248, S249, E250, P251, A252, S253, N254, N255, P256, K257, K258, A259, G260, V261, Y262, T266, Y267, V268, T269, V270, E271, I272, K273, G274, P275, G276, P277, G278, K279, A280, Q281, L282, A283, K284, A285, V286, K287, N288, P289, A290, P291, I292, S293, G294, P295, G296, P297, G298, D299, V300, L301, I302, S303, N304, M305, S306 | 63 | 0.755 |
| **2** | A359, A360, Y361, S362, V363, A364, G365, V366, Y367, N368, G369, K370, A371, A372, Y373, A374, P375, Q376, D377, K378, P379, A380, E381, A382, A383, A384, Y385, A386, P387, A388, A389, Q390, A391 | 33 | 0.747 |
| **3** | A55, T58, K59, L60, Q61, E62, D63, L64, P65, E66, Q67, L68, T69, E70, L71, R72, E73, K74, F75, T76, A77, E78, E79, L80, R81, K82, A83, A84, E85, G86, Y87, L88, E89, G138, V140, A141, S142, Q143, T144, R145, A146, V147, G148, E149, R150, A151, A152, K153, L154, V155, G156, I157, E158, L159, P160, K161 | 56 | 0.745 |
| **4** | K162, A163, A164, P165, A166, K167, K168, A169, A170, P171, A172, K173, K174, A175, A176, P177, A178, K179, K180, A181, K184 | 21 | 0.737 |
| **5** | M1, A2, E3, N4, S5, N6, I7, D8, D9, I10, K11, A220, A221, K222, P223, V224, A225, V226, R227, T228, H229 | 21 | 0.641 |
| **6** | G440, A441, A443, A444, H445, H446, H447, H448, H449, H450 | 10 | 0.626 |
| **7** | A424, Q425, D426, S427, A428, P429, A430, K432, A433, A434, A436, V437 | 12 | 0.576 |
| **8** | K338, P339, V340 | 3 | 0.553 |
| **9** | R231, N232, V234, I235, L236, P237, E238, K240 | 8 | 0.535 |
