# Supplementary material for: In silico design of a multi-epitope vaccine against Cryptosporidium parvum using structural and immunoinformatics approaches
Source: PLoS One. 2025 Nov 18;20(11):e0334754. doi: 10.1371/journal.pone.0334754 (PMC12626319; doi:10.1371/journal.pone.0334754)
Supplement: S5 Table — (DOCX) [file pone.0334754.s010.docx]

**S5 Table.** Worldwide population coverage assessment of the chosen HTL and CTL epitopes.

| **Country** | **Coverage** |
| --- | --- |
| Central Africa | 70.09 |
| East Africa | 83.05 |
| East Asia | 90.69 |
| Europe | 98.91 |
| North Africa | 89.13 |
| North America | 96.46 |
| Northeast Asia | 88.77 |
| Oceania | 76.69 |
| South Africa | 86.47 |
| South America | 77.66 |
| South Asia | 90.45 |
| Southeast Asia | 84.57 |
| Southwest Asia | 87.94 |
| West Africa | 85.59 |
| West Indies | 94.07 |
| World | 95.92 |
