# Supplementary material for: In silico design of a multi-epitope vaccine against Cryptosporidium parvum using structural and immunoinformatics approaches
Source: PLoS One. 2025 Nov 18;20(11):e0334754. doi: 10.1371/journal.pone.0334754 (PMC12626319; doi:10.1371/journal.pone.0334754)
Supplement: Attachment — Submitted filename: comment.pdf [file pone.0334754.s014.pdf]

The manuscript entitled **"In silico design of a multi-epitope vaccine against *Cryptosporidium parvum* using a structural and immunoinformatics approach"** offers a comprehensive computational framework for the rational development of a multi-epitope subunit vaccine targeting *C. parvum*. The topic is highly pertinent given the global health relevance of cryptosporidiosis, and the study reflects a solid application of contemporary approaches in vaccinology and immunoinformatics. The methodology is coherent and appropriately selected, demonstrating the potential of in silico techniques in accelerating vaccine design.

The authors have effectively utilized various immunoinformatics tools to identify potential antigens and epitopes, construct a chimeric vaccine sequence, and evaluate its structural and immunological properties through docking, immune simulation, and codon optimization. The manuscript is generally clear and flows logically from one section to the next.

A **minor revision** would enhance the overall clarity and impact of the work. Specifically, some methodological descriptions could benefit from additional detail to improve reproducibility. Additionally, a brief discussion on the potential limitations of the approach (e.g., reliance on predictive models without experimental validation) would provide a more balanced perspective.

Comments:

1. Describe the impact of cryptosporidiosis on global health and livestock economics.

2. What challenges are associated with current treatment options for cryptosporidiosis, particularly in immunocompromised individuals?

3. Explain the role of *in silico* tools in the initial screening and refinement of epitopes for vaccine design.

4. Why was LprA chosen as an adjuvant, and what is its expected immunological role in the MESV construct?

5. What are the key physicochemical features considered essential for a successful vaccine construct, and how were these evaluated?

6. How does the use of trRosetta and GalaxyRefine2 contribute to the structural validity of the vaccine candidate?

7. Briefly explain the significance of the molecular docking study involving TLR2 and TLR4.

8. What is the purpose of using GROMACS and PCA in the vaccine evaluation pipeline?
