# Supplementary material for: In silico design of a multi-epitope vaccine against Cryptosporidium parvum using structural and immunoinformatics approaches
Source: PLoS One. 2025 Nov 18;20(11):e0334754. doi: 10.1371/journal.pone.0334754 (PMC12626319; doi:10.1371/journal.pone.0334754)
Supplement: Attachment — Submitted filename: Response_to_Reviewers-final.pdf [file pone.0334754.s015.pdf]

Response to Reviewers

We sincerely thank all the reviewers for their valuable time and insightful comments. Their constructive feedback has greatly helped us to improve the quality and clarity of the manuscript. We have carefully revised the manuscript in response to each comment. The changes have been highlighted in yellow in the revised manuscript, and line numbers have been provided wherever applicable for ease of reference.

Reviewer 1

Comments:

Comment 1. The target antigens should be explicitly mentioned in the abstract for clarity and completeness.

Response: We appreciate this suggestion. We have revised the abstract to include the specific proteins used, Cp15, Cp23, and CpP2, as the target antigens (Line no. 28-29).

Comment 2. The Introduction section lacks a comprehensive overview of the currently available therapeutic and prophylactic strategies against *C. parvum*. A critical discussion of their strengths and limitations would help position the current study within the broader research context.

Response: We have substantially revised the introduction to include a detailed overview of current therapies (e.g., Nitazoxanide) and their limitations, as well as the lack of effective vaccines. This provides a stronger rationale for our immunoinformatics approach (line no. 78-90).

3. References 19–22 indicate that similar in-silico vaccine designs have already been reported. The authors should clearly articulate the novelty of their approach and justify how this work adds to the existing literature.

Response: Thank you for the comment. While References 19–22 report wet-lab studies involving Cp15, Cp23, and CpP2, they focus on individual or fusion protein-based vaccines and do not employ a multi-epitope subunit vaccine design. Our study is novel in integrating these experimentally validated antigens into a structure-based MESV using immunoinformatics tools, incorporating CTL, HTL, B-cell, and IFN-γ epitopes, and validating the construct through docking, immune simulation, and population coverage analysis. This approach offers a new direction for *C. parvum* vaccine development.

Comment 4. The same proteins Cp15, Cp23, and CpP2 have been previously used in related studies. Without additional experimental validation or innovative methodological contributions, the manuscript lacks sufficient novelty.

Response: Thank you for the comment. While Cp15, Cp23, and CpP2 have been individually validated in wet-lab studies such as Cp15-DNA vaccination in goats [1], Cp15–23 fusion protein

in mice [2], and CpP2-DNA immunization in IL-12 KO mice [3], they have not been explored collectively in a multi-epitope subunit vaccine design. Our study is novel in integrating these experimentally validated antigens using a structure-based immunoinformatics approach, incorporating CTL, HTL, B-cell, and IFN-γ–inducing epitopes, and performing molecular docking, immune simulation, and population coverage analysis. This computational MESV design represents a new and rational step toward vaccine development against *C. parvum*.

References:

1. Sagodira, Serge, et al. "Protection of kids against Cryptosporidium parvum infection after immunization of dams with CP15-DNA." Vaccine 17.19 (1999): 2346-2355.
2. Liu, K., et al. "Divalent Cp15–23 vaccine enhances immune responses and protection against Cryptosporidium parvum infection." Parasite immunology 32.5 (2010): 335-344.
3. Benitez, Alvaro, et al. "Evaluation of DNA encoding acidic ribosomal protein P2 of Cryptosporidium parvum as a potential vaccine candidate for cryptosporidiosis." Vaccine 29.49 (2011): 9239-9245.

Comment 5. A dedicated discussion on the advantages and limitations of in-silico vaccine design, supported by recent literature, is essential to provide a balanced perspective.

Response: We thank the reviewer for this valuable suggestion. We have now included a dedicated section in the Discussion that outlines both the advantages and limitations of in-silico vaccine design, supported by recent literature (Line no. 568-577).

6. The manuscript does not reference any studies that demonstrate the efficacy of multi-epitope vaccines specifically against parasitic infections. Including such references would strengthen the rationale for the approach.

Response: Relevant references on multi-epitope vaccines for parasites such as *Plasmodium falciparum*, *Leishmania donovani*, *Fasciolopsis buski*, and *Toxoplasma gondii* have been added to the discussion to support the relevance of our strategy (Line no. 516-519).

7. The strain of *C. parvum* from which the protein sequences were retrieved is not specified. This information is critical for reproducibility and comparative evaluation.

Response: The protein sequences used in this study were retrieved from UniProt, with the following identifiers: Cp15 (Q23728), Cp23 (Q8ITU5), and CpP2 (Q9U553). According to UniProt, all three sequences correspond to the *Cryptosporidium parvum* Iowa II strain, which is commonly used in experimental studies and sequence repositories. We have now included this information in the Methods section to ensure reproducibility and facilitate comparative evaluation (Line no. 121).

8. The use of the LprA adjuvant (244 amino acids) in a final construct of 450 amino acids suggests a disproportionately low contribution from B-cell, CTL, and HTL epitopes. This raises concerns about the immunogenic balance of the construct and whether observed immune responses would be attributable to the adjuvant or the selected epitopes.

Response: We appreciate the reviewer’s concern regarding the proportion of the LprA adjuvant relative to the total length of the MESV construct. While LprA constitutes approximately half of the 450-amino-acid vaccine, its role is to enhance antigen presentation and stimulate TLR2-mediated innate immune signaling rather than to directly provide specific B-cell or T-cell epitopes. The selected CTL, HTL, and B-cell epitopes, though shorter in length, are highly immunogenic and were carefully chosen based on antigenicity, conservancy, and predicted binding affinity to human HLA molecules. Importantly, in silico immune simulations indicated robust humoral and cellular responses, including elevated levels of B cells, memory T cells, and cytokines (IFN-γ, IL-2, IL-10, IL-12), suggesting that both the adjuvant and the epitopes contribute synergistically to the overall immune response.

9. The rationale for performing in-silico cloning is unclear in the absence of downstream experimental validation. This section appears tangential and may be omitted or better justified.

Response: We thank the reviewer for raising this point. The *in silico* cloning and codon optimization analyses were included to evaluate the feasibility of recombinant expression in *E. coli* and to anticipate potential challenges in protein production. While downstream experimental validation is pending, these analyses provide a preliminary assessment of translational efficiency and guide future experimental planning. We have clarified this rationale in the Discussion section (Line no. 564-567).

10. The Discussion section largely reiterates the results without offering critical analysis or biological interpretation. It requires substantial revision to provide meaningful insights and contextual relevance.

Response: We appreciate the reviewer’s observation regarding the Discussion section. We have revised the Discussion to move beyond mere repetition of results and to provide a more critical analysis of the findings. In the revised version, we contextualize the computational outcomes within the broader biological and immunological framework of *C. parvum* infection, highlighting the relevance of selected epitopes, linkers, and adjuvants in eliciting both humoral and cellular immunity. We also discuss the translational implications of population coverage, mRNA secondary structure predictions, and immune simulation results, as well as the limitations of *in silico* approaches and the need for subsequent *in vitro* and *in vivo* validation. These revisions aim to provide meaningful insights and interpretative depth, addressing the reviewer’s concern regarding biological significance.

11. Figure quality is very poor.

Response: Thank you for your feedback. We would like to clarify that high-resolution images were uploaded during the submission process. However, due to the system's formatting, the images may appear compressed within the main PDF file. To view the figures in their original quality, kindly use the “Click here to access/download: Figure” links provided in the submission system under each figure (e.g., Figure; Fig.). We appreciate your understanding.

Reviewer 2

Major Comments

1.The author drafted the manuscript clearly.

2. I encourage the authors to provide more details about how serious the problem is. Including statistics on the mortality rate, if available, would help show the importance of this study.

Response:

We thank the reviewer for this valuable suggestion. To better emphasize the global significance of cryptosporidiosis, we have revised the introduction to include specific prevalence data in both livestock and humans. This includes infection rates ranging from 11.7% to 78% in pre-weaned calves, and human prevalence rates of up to 31.5% in low-income regions. We have also referenced the Global Enteric Multicenter Study (GEMS), which estimates nearly 202,000 child deaths annually in sub-Saharan Africa and South Asia. Additionally, we noted that *C. parvum* accounted for over 96% of foodborne morbidity cases between 2010 and 2020, further underlining its public health and economic impact. These additions underscore the urgency for effective vaccine development. The revised text has been highlighted in yellow from lines 68–77 in the manuscript.

3. Please provide an overview of the current treatment options available for Cryptosporidium parvum infections, including their associated side effects. Additionally, explain the significance of your study in addressing the limitations of these treatments and advancing vaccine development.

Response:

We thank the reviewer for the insightful comment. We have revised the introduction to provide an overview of current treatments for *Cryptosporidium parvum*. Management mainly involves supportive care and nitazoxanide, the only FDA-approved drug, which shows limited efficacy in immunocompetent individuals and is ineffective in immunocompromised patients. Other agents (paromomycin, azithromycin, clofazimine) have produced inconsistent results, and nitazoxanide is associated with gastrointestinal side effects and poor tolerability. These limitations underscore the urgent need for preventive strategies. Our study addresses this gap by designing a multi-epitope subunit vaccine targeting immunodominant *C. parvum* antigens to elicit robust mucosal and systemic immunity while avoiding risks linked to live or whole-parasite vaccines (Line no. 78-90).

4. What is the worldwide impact or burden of this problem?

Response:

We thank the reviewer for this insightful question. The worldwide impact and burden of cryptosporidiosis have been addressed in the manuscript. Specifically, we have included details on human prevalence, child mortality, livestock prevalence, neonatal mortality, and foodborne morbidity. These points are covered in the manuscript from line numbers 68-77.

5. The presented 3.5. Global population coverage and epitope conservation explores how effectively the predicted epitopes can elicit immune responses across diverse human populations based on HLA diversity.

Response:

We thank the reviewer for this comment. The section on global population coverage and epitope conservation was included to assess how effectively the predicted T-cell epitopes can stimulate immune responses across diverse human populations, considering the high polymorphism of HLA molecules. Our analysis ensured that selected epitopes bind to multiple HLA supertypes, maximizing coverage across 16 global regions, with Europe showing the highest coverage (98.91%) and Central Africa the lowest (70.09%) (Fig. 5; Supplementary Table 5). Conservation analysis further confirmed that most epitopes from CP15, CP23, and CpP2 proteins are highly conserved across strains, supporting their potential to induce broad-spectrum immune protection. Together, these analyses demonstrate that the predicted epitopes are likely to be effective in eliciting immune responses across diverse populations.

6. The images appear to be unclear and may require improved quality for better visibility.

Response: Thank you for your feedback. We would like to clarify that high-resolution images were uploaded during the submission process. However, due to the system's formatting, the images may appear compressed within the main PDF file. To view the figures in their original quality, we kindly suggest downloading them using the “Click here to access/download: Figure” links available at the top right-hand corner of the PDF file. Additionally, the population coverage image has been replaced with a higher-resolution version to enhance readability. We appreciate your understanding.

7. Can the authors explain why they did not use the newer software tools available for this analysis? The tools they used are popular, but using the latest ones might add more interest.

Response:

We thank the reviewer for this valuable suggestion. We acknowledge that several newer tools have been developed for population coverage and conservancy analysis. However, in our study, we used the IEDB population coverage tool, MEGA v7.0, and WebLogo v3.7.9 because they are widely accepted, validated, and extensively cited in the literature, ensuring reproducibility and comparability with previous studies. Importantly, the IEDB population coverage tool remains the standard and most reliable resource for HLA-based coverage analysis, as it integrates curated data directly from IEDB. Similarly, MEGA and WebLogo have been consistently used for sequence alignment and conservation analysis, offering robust and transparent results.

We agree that newer platforms may offer additional features; however, our selection was based on reliability, accessibility, and acceptance in immunoinformatics studies.

8. I encourage the authors to approach their research from a unique perspective and clearly emphasize the novelty of their study.

Response:

We thank the reviewer for this suggestion. In the revised manuscript, we have emphasized the novelty of our study by highlighting several key aspects: the use of experimentally validated *C. parvum* antigens rather than hypothetical proteins, the integration of PCA and MM-GBSA analyses to evaluate dynamic stability and binding energetics, the assessment of global population coverage, which was predicted to reach 95.92% across 16 regions, and the evaluation of mRNA secondary structure to infer translational efficiency. These elements collectively distinguish our work from previous MESV designs and provide both structural and translational insights, underscoring the unique contribution of our study to the field of cryptosporidiosis vaccine development.

9. It would be helpful to clearly highlight the key differences and novel contributions of your study in comparison to this existing work (An immunoinformatics approach for design and validation of multi-subunit vaccine against Cryptosporidium parvum) see this paper mostly like your study.

Response:

We appreciate the reviewer's suggestion to clarify the novel contributions of our study relative to the existing work. While the referenced study employed an immunoinformatics approach to design a multi-subunit vaccine against *C. parvum*, our study differs in several important aspects:

Use of experimentally validated antigens: Unlike the previous work, which relied largely on predicted or hypothetical proteins, we focused on experimentally verified antigens (Cp15, Cp23, CpP2) that play established roles in parasite invasion and host immune recognition.

Rigorous structural and energetic validation: We incorporated PCA and MM-GBSA analyses to evaluate the dynamic stability and binding energetics of the vaccine construct, providing a deeper mechanistic understanding of epitope-receptor interactions.

Global population coverage analysis: Our MESV was assessed for potential coverage across 16 regions, with a predicted 95.92% coverage, demonstrating broad applicability across diverse ethnic and geographic groups.

Translational insights: We evaluated the mRNA secondary structure and performed in silico codon optimization, providing preliminary insights into translational efficiency and recombinant expression feasibility, which were not explored in the previous study.

Collectively, these methodological enhancements and translational considerations distinguish our work from previous designs, highlighting both its novelty and potential practical relevance in *C. parvum* vaccine development (Line no. 545-557).

Reviewer 3

Comments & Questions:

1. What specific thresholds were applied for antigenicity, allergenicity, and toxicity during epitope screening?

Response: Antigenicity of epitopes was assessed using VaxiJen v2.0 with a threshold of 0.4. Allergenicity was evaluated using AllerTOP v2.0, with non-allergenic epitopes selected. Toxicity was predicted via ToxinPred, and only non-toxic peptides were included in downstream analyses. These thresholds are widely cited in immunoinformatics studies.

2. Why was LprA specifically selected as the TLR2 agonist over other known adjuvants?

Response: LprA was selected because it is a well-characterized TLR2 agonist known to enhance Th1-biased immune responses, which are crucial for protection against intracellular pathogens such as *C. parvum*. Other adjuvants were considered, but LprA provides both safety and efficacy in subunit vaccine design [line no. 531-535].

3. Nitazoxanide is mentioned as ineffective in immunocompromised patients. Are there any other drugs under investigation that should be noted?

Response: Several investigational agents, such as paromomycin, clofazimine, and azithromycin, have been evaluated for cryptosporidiosis, but most show limited efficacy in immunocompromised patients. This reinforces the need for preventive vaccines rather than relying solely on therapeutics (Line no. 78-86).

4. The authors mention safety concerns with live attenuated vaccines. Could you briefly comment on any documented adverse events in immunocompromised individuals?

Response: We thank the reviewer for this point. Live attenuated vaccines can pose safety risks in immunocompromised individuals, including uncontrolled infection or prolonged pathogen shedding. Such risks highlight the need for safer alternatives, like multi-epitope subunit vaccines. As noted, current treatments like nitazoxanide also cause adverse effects, including gastrointestinal disturbances and headache, underscoring the need for tolerable prophylactic strategies (Line no. 82-86).

5. Did the authors consider targeting HLA population coverage in endemic regions (e.g., Africa or Asia) during epitope selection?

Response: Yes, population coverage analysis included 16 global regions, with particular attention to Africa and Asia, where *C. parvum* burden is highest. This ensured that selected T-cell epitopes provide broad immunogenicity in endemic populations.

6. You mention lack of transmembrane helices — was any signal peptide prediction also performed (e.g., using SignalP)? This is relevant for identifying secretory/extracellular proteins.

Response: In addition to confirming the absence of transmembrane helices, we also performed signal peptide prediction using SignalP 6.0. However, none of the selected antigens were found to contain signal peptides, and therefore, this result was not included in the manuscript.

7. You mention PADREE. Did you mean the PADRE sequence (universal helper T-cell epitope)? Please correct spelling to avoid confusion.

Response: We apologize for the typographical error. The correct term is PADRE (Pan HLA-DR epitope), a universal helper T-cell epitope, and this has been corrected throughout the manuscript (Line no. 159).

8. Why was trRosetta selected over AlphaFold2 or ColabFold? These offer superior modeling and are freely accessible.

Response: trRosetta was selected for its accuracy in predicting tertiary structures from sparse sequence alignments and for its efficiency with multiple proteins. While AlphaFold2 and ColabFold are excellent tools, trRosetta provided robust models compatible with downstream docking and MD simulations in our workflow.

9. What criteria were used to interpret the flexibility plots? Did high flexibility regions overlap with epitope regions?

Response: Flexibility plots (B-factor/mobility profiles) were interpreted such that regions with higher flexibility may be more exposed and accessible for immune recognition. Many predicted epitopes overlapped with moderately flexible regions, supporting their potential immunogenicity.

10. AGGRESCAN 3D was mentioned — were any aggregation-prone regions found? Were adjustments made accordingly?

Response: Aggregation-prone regions were identified using AGGRESCAN 3D. Only minor hotspots were observed, which were not located within the predicted epitope regions, so no adjustments were necessary for MESV construct design.

11. If linear B-cell epitopes were already predicted in section 2.2, what was the rationale for also using conformational prediction here? Were both types used in MESV, or only one?

Response: We thank the reviewer for this important point. Both linear and conformational B-cell epitopes were predicted because they represent complementary aspects of antigen recognition. Linear epitopes are useful for identifying continuous amino acid stretches, while conformational epitopes capture the discontinuous regions that arise after protein folding, which are often critical for antibody binding. However, only the linear B-cell epitopes were included in the final MESV construct, while conformational predictions were used to validate structural accessibility and immunological relevance.

Reviewer 4

Specific comments:

1. Explain how the transmission of Cryptosporidium parvum contributes to its persistence in both human and animal populations.

Response: *Cryptosporidium parvum* is transmitted primarily via the fecal-oral route through contaminated water, food, and surface, facilitating widespread zoonotic and anthroponotic transmission. Its ability to infect a broad range of hosts, including both humans and livestock, combined with the environmental resilience of its oocytes, enables sustained transmission cycles across species. This dual host reservoir and waterborne stability significantly contribute to its persistence in endemic areas.

2. What role do thick-walled and thin-walled oocysts play in the life cycle of *C. parvum*, and how does this relate to disease chronicity?

Response: Thick-walled oocysts are excreted into the environment and are responsible for transmission to new hosts, while thin-walled oocysts remain within the host's intestine and facilitate autoinfection. The presence of thin-walled oocysts enables continual reinfection of host intestinal cells without environmental exposure, promoting persistent or chronic infection, especially in immunocompromised individuals.

3. Justify the choice of Cp15, Cp23, and CpP2 as candidate antigens in the MESV design.

Response: Cp15, Cp23, and CpP2 are well-characterized surface or secretory proteins involved in host-cell attachment and invasion. They are highly conserved, immunodominant, and have been shown to elicit strong antibody and T-cell responses in both natural infections and vaccine studies. Their extracellular localization makes them readily accessible to the host immune system, justifying their inclusion in a multi-epitope vaccine (Line no. 100-106).

4. Describe how the use of signal peptide and transmembrane helix predictions contributes to antigen selection.

Response: We appreciate the reviewer's point. Prediction of signal peptides and transmembrane helices is valuable in antigen selection, as it helps identify proteins that are secreted or surface-exposed and thus accessible to host immune responses, while avoiding those with multiple transmembrane domains. In our analysis, none of the shortlisted proteins contained transmembrane helices (as already reported in the manuscript) or signal peptides. Since the signal peptide prediction did not yield positive results, we did not include this in the final manuscript.

5. Why was it necessary to perform allergenicity and human similarity screening for selected protein sequences? What structural refinement criteria have been selected while designing the vaccine?

Response: Allergenicity screening ensures the designed vaccine does not trigger allergic reactions, while human similarity screening avoids autoimmunity by excluding epitopes with high homology to human proteins. These filters enhance the safety profile of the vaccine by minimizing potential

adverse immune responses in the host. For structural refinement of the designed vaccine construct, we used the GalaxyRefine2 server, which performs iterative structure relaxation through side-chain repacking and molecular dynamics simulations. The refinement criteria include optimization of local geometry, removal of steric clashes, and improvement of overall stereochemical quality, thereby generating a more stable and biologically relevant 3D vaccine model.

6. What are the advantages of using multiple servers (e.g., ABCpred and BepiPred-3.0) for B-cell epitope prediction?

Response: Each prediction server uses distinct algorithms and training datasets, leading to variability in predicted epitopes. Using multiple tools enhances prediction reliability by identifying consensus epitopes, thus reducing false positives and improving the overall confidence in selected B-cell epitope regions.

7. Explain the significance of using linkers such as AAY, GPGPG, and KK in the final MESV construct.

Response: Linkers serve multiple roles in vaccine design: AAY promotes proteasomal processing for cytotoxic T-cell epitopes. GPGPG maintains conformational independence between helper T-cell epitopes, enhancing their presentation by MHC II molecules. KK linkers improve solubility and flexibility, particularly for B-cell epitopes. Together, these linkers preserve immunogenicity, enhance epitope processing, and prevent junctional epitope formation.

8. Discuss the rationale for including the PADRE sequence and a His-tag in the vaccine design.

Response: The PADRE (Pan HLA-DR-binding epitope) sequence enhances helper T-cell activation by promoting broader MHC class II coverage, especially in genetically diverse human populations. The His-tag facilitates recombinant protein purification through affinity chromatography, streamlining downstream expression and purification steps.

9. What is the significance of docking in this study?

Response: The significance of docking in this study lies in evaluating the interaction between the designed multi-epitope vaccine and Toll-like receptors (TLRs). Since TLRs, particularly TLR2 and TLR4, play a central role in initiating innate immune responses by recognizing pathogen-associated molecular patterns, docking analysis provides insights into whether the designed vaccine can effectively bind and stimulate these receptors. A strong and stable interaction suggests the potential of the construct to trigger downstream signaling pathways, leading to the activation of adaptive immunity. Thus, docking serves as a crucial *in silico* validation step to predict the immunogenic potential and functional relevance of the vaccine candidate before moving to experimental studies.

10. What is the importance of predicting both linear and conformational B-cell epitopes in vaccine design?

Response: Linear epitopes are recognized by B cells in their native form, while conformational epitopes are recognized only when the protein retains its 3D structure. Predicting both types ensure

comprehensive B-cell immune stimulation, increasing the likelihood of generating neutralizing antibodies that recognize the native antigen conformation in vivo.

Reviewer 5

Comments:

1. Describe the impact of cryptosporidiosis on global health and livestock economics.

Response: Cryptosporidiosis is a major cause of diarrheal disease in both humans and animals, particularly in young children, immunocompromised individuals, and neonatal livestock. It contributes significantly to global child morbidity and mortality, especially in low-resource settings. In livestock, it causes growth retardation, reduces productivity, results in economic loss due to veterinary care, and affects the market value of affected animals. Its zoonotic potential exacerbates public health and agricultural burdens.

2. What challenges are associated with current treatment options for cryptosporidiosis, particularly in immunocompromised individuals?

Response: Thank you for raising this important point. Current treatment options for cryptosporidiosis are limited, as nitazoxanide, the only FDA-approved drug, shows reduced or no efficacy in immunocompromised individuals, such as HIV/AIDS patients or transplant recipients. Additionally, most available drugs act only symptomatically, do not completely eradicate the parasite, and face challenges of resistance development. This underlines the urgent need for alternative strategies, such as vaccines, to provide long-term protection.

3. Explain the role of in silico tools in the initial screening and refinement of epitopes for vaccine design.

Response: We appreciate this question. In silico tools allow rapid and high-throughput screening of potential epitopes based on antigenicity, allergenicity, toxicity, and population coverage. These tools reduce the cost and time associated with experimental screening. Furthermore, refinement algorithms help prioritize epitopes that are conserved, immunogenic, and capable of eliciting both humoral and cellular immune responses, thereby ensuring the construct is both safe and effective.

4. Why was LprA chosen as an adjuvant, and what is its expected immunological role in the MESV construct?

Response: Thank you for asking this. LprA, a lipoprotein from Mycobacterium tuberculosis, was selected as an adjuvant because it is a well-known TLR2 agonist that enhances innate immune recognition. Its inclusion in the MESV construct is expected to improve antigen processing and presentation, promote Th1-biased responses, and ultimately boost the immunogenicity of the vaccine. This helps overcome the otherwise weak immunostimulation often associated with subunit vaccines.

5. What are the key physicochemical features considered essential for a successful vaccine construct, and how were these evaluated?

Response: Key physicochemical features include molecular weight, theoretical pI, stability index, aliphatic index, hydropathicity (GRAVY), and solubility. These parameters are essential for determining whether the construct will be stable, soluble, and suitable for large-scale production. We evaluated these features using the ProtParam and SOLpro servers, which confirmed that the designed MESV is stable, soluble, and has favorable biochemical characteristics required for an effective vaccine candidate.

6. How does the use of trRosetta and GalaxyRefine2 contribute to the structural validity of the vaccine candidate?

Response: Thank you for this insightful query. trRosetta was used for the initial 3D structure prediction of the MESV construct based on deep-learning guided restraints, ensuring a reliable starting model. GalaxyRefine2 was then employed for iterative refinement through side-chain repacking and molecular dynamics relaxation, improving stereochemistry, reducing steric clashes, and enhancing overall structural quality. Together, these steps ensure a structurally valid and biologically relevant model.

7. Briefly explain the significance of the molecular docking study involving TLR2 and TLR4.

Response: Docking with TLR2 and TLR4 is significant because these receptors play central roles in innate immune activation. Evaluating the binding affinity and stability of interactions between the MESV and these receptors helps predict whether the vaccine can effectively trigger innate signaling pathways. Strong binding with TLRs indicates that the construct has the potential to initiate downstream cytokine production and adaptive immune responses, thereby validating its immunogenic potential.

8. What is the purpose of using GROMACS and PCA in the vaccine evaluation pipeline?

Response: Thank you for this question. GROMACS was used to perform molecular dynamics simulations, allowing us to study the stability, conformational changes, and dynamic behavior of the MESV–TLR complexes under physiological conditions. Principal Component Analysis (PCA) was applied to extract dominant motion patterns and evaluate essential dynamics, providing deeper insights into conformational flexibility and stability. Together, these analyses ensure that the vaccine–receptor complexes remain stable and biologically viable.
