# Supplementary material for: In silico design of a multi-epitope vaccine against Cryptosporidium parvum using structural and immunoinformatics approaches
Source: PLoS One. 2025 Nov 18;20(11):e0334754. doi: 10.1371/journal.pone.0334754 (PMC12626319; doi:10.1371/journal.pone.0334754)
Supplement: Attachment — Submitted filename: PONE-D-25-36025_Dipti_review_comments.docx [file pone.0334754.s011.docx]

**In silico design of a multi-epitope vaccine against Cryptosporidium parvum using structural and immunoinformatic approach**

**Jeong Ho Hwang,et al,**

**Major Comments**

1.The author drafted the manuscript clearly.

2. I encourage the authors to provide more details about how serious the problem is. Including statistics on the mortality rate, if available, would help show the importance of this study.

3. Please provide an overview of the current treatment options available for Cryptosporidium parvum infections, including their associated side effects. Additionally, explain the significance of your study in addressing the limitations of these treatments and advancing vaccine development.

4. What is the worldwide impact or burden of this problem?

5. The presented 3.5. Global population coverage and epitope conservation explores how effectively the predicted epitopes can elicit immune responses across diverse human populations based on HLA diversity.

6. The images appear to be unclear and may require improved quality for better visibility.

7. Can the authors explain why they did not use the newer software tools available for this analysis? The tools they used are popular, but using the latest ones might add more interest.

8.I encourage the authors to approach their research from a unique perspective and clearly emphasize the novelty of their study.

9. It would be helpful to clearly highlight the key differences and novel contributions of your study in comparison to this existing work (An immunoinformatics approach for design and validation of multi-subunit vaccine against Cryptosporidium parvum) see this paper mostly like your study.

**Decision: The manuscript is accepted after carefully addressing all the comments**
