# Supplementary material for: In silico design of a multi-epitope vaccine against Cryptosporidium parvum using structural and immunoinformatics approaches
Source: PLoS One. 2025 Nov 18;20(11):e0334754. doi: 10.1371/journal.pone.0334754 (PMC12626319; doi:10.1371/journal.pone.0334754)
Supplement: Attachment — Submitted filename: Review report_PONE-D-25-36025.docx [file pone.0334754.s012.docx]

**Review report**

The manuscript titled **“In silico design of a multi-epitope vaccine against *Cryptosporidium parvum* using a structural and immunoinformatics approach”** presents a comprehensive computational strategy for developing a multi-epitope subunit vaccine (MESV) targeting *Cryptosporidium parvum*. The study integrates several advanced immunoinformatics techniques, including antigenicity prediction, molecular docking, immune simulation, and codon optimization. The work is timely, methodologically robust, and represents a valuable contribution to the field of rational vaccine design. However, minor revisions are recommended to enhance clarity and scientific rigor prior to publication.

**Comments & Questions:**

1. What specific thresholds were applied for antigenicity, allergenicity, and toxicity during epitope screening?

2. Why was LprA specifically selected as the TLR2 agonist over other known adjuvants?

3. Nitazoxanide is mentioned as ineffective in immunocompromised patients. Are there any other drugs under investigation that should be noted?

4. The authors mention safety concerns with live attenuated vaccines. Could you briefly comment on any documented adverse events in immunocompromised individuals?

5. Did the authors consider targeting HLA population coverage in endemic regions (e.g., Africa or Asia) during epitope selection?

6. You mention lack of transmembrane helices — was any signal peptide prediction also performed (e.g., using SignalP)? This is relevant for identifying secretory/extracellular proteins.

7. You mention PADREE. Did you mean the PADRE sequence (universal helper T-cell epitope)? Please correct spelling to avoid confusion.

8. Why was trRosetta selected over AlphaFold2 or ColabFold? These offer superior modeling and are freely accessible.

9. What criteria were used to interpret the flexibility plots? Did high flexibility regions overlap with epitope regions?

10. AGGRESCAN 3D was mentioned — were any aggregation-prone regions found? Were adjustments made accordingly?

11. If linear B-cell epitopes were already predicted in section 2.2, what was the rationale for also using conformational prediction here? Were both types used in MESV, or only one?
