# Supplementary material for: In silico design of a multi-epitope vaccine against Cryptosporidium parvum using structural and immunoinformatics approaches
Source: PLoS One. 2025 Nov 18;20(11):e0334754. doi: 10.1371/journal.pone.0334754 (PMC12626319; doi:10.1371/journal.pone.0334754)
Supplement: Attachment — Submitted filename: renamed_1264f.docx [file pone.0334754.s013.docx]

**Reviewer’s Comments**

The manuscript entitled **“In silico design of a multi-epitope vaccine against Cryptosporidium parvum using a structural and immunoinformatics approach”** presents a relevant and timely contribution to the field of computational vaccine design. The study employs a variety of immunoinformatics tools to develop a multi-epitope subunit vaccine (MESV) targeting C. parvum, a pathogen of significant public health concern. The integration of antigenicity prediction, structural modeling, immune simulation, and codon optimization reflects a commendable effort toward a comprehensive in silico vaccine design pipeline.

However, in its current form, the manuscript would benefit from **major revisions** to improve clarity, coherence, and scientific rigor. While the methodology is broadly appropriate, several sections would benefit from **clearer explanation, more detailed descriptions, and stronger justification of key design choices** (e.g., antigen selection, docking protocol, and structural refinement). Additionally, the inclusion of comparative analyses or benchmarking with existing approaches would strengthen the overall impact of the study.

To enhance the reproducibility and interpretability of the findings, I recommend addressing the points outlined in the detailed review, including clarification of computational workflows, improved rationale for methodological decisions, and more thorough discussion of the biological relevance and potential limitations of the proposed vaccine construct.

**Specific comments:**

1. Explain how the transmission of *Cryptosporidium parvum* contributes to its persistence in both human and animal populations.

2. What role do thick-walled and thin-walled oocysts play in the life cycle of C. parvum, and how does this relate to disease chronicity?

3. Justify the choice of Cp15, Cp23, and CpP2 as candidate antigens in the MESV design.

4. Describe how the use of signal peptide and transmembrane helix predictions contributes to antigen selection.

5. Why was it necessary to perform allergenicity and human similarity screening for selected protein sequences? What structural refinement criteria have been selected while designing the vaccine?

6. What are the advantages of using multiple servers (e.g., ABCpred and BepiPred-3.0) for B-cell epitope prediction?

7. Explain the significance of using linkers such as AAY, GPGPG, and KK in the final MESV construct.

8. Discuss the rationale for including the PADRE sequence and a His-tag in the vaccine design.

9. What is the significance of docking in this study?

10. What is the importance of predicting both linear and conformational B-cell epitopes in vaccine design?
