# Supplementary material for: In silico design of a multi-epitope vaccine against Cryptosporidium parvum using structural and immunoinformatics approaches
Source: PLoS One. 2025 Nov 18;20(11):e0334754. doi: 10.1371/journal.pone.0334754 (PMC12626319; doi:10.1371/journal.pone.0334754)
Supplement: Attachment — Submitted filename: Comments to upload.docx [file pone.0334754.s016.docx]

**Comments:**

The revised manuscript entitled "***In silico design of a multi-epitope vaccine against Cryptosporidium parvum using structural and immunoinformatics approaches***" has addressed all the concerns raised in the previous review. The authors have provided comprehensive responses and incorporated the necessary revisions to improve clarity and scientific rigor. The methodology, results, and discussion are now well-structured, and the conclusions are supported by the data presented. Given these improvements, I recommend the manuscript for acceptance in its current form.
